# The abnormal lipid profile in obesity and coronary heart disease (CHD) in Pakistani subjects

**DOI:** 10.1186/s12944-020-01248-0

**Published:** 2020-04-14

**Authors:** Shabana, Saleem Ullah Shahid, Sumbal Sarwar

**Affiliations:** Department of Microbiology and Molecular Genetics, University of the Punjab, Lahore, 54690 Pakistan

**Keywords:** Dyslipidemia, Obesity, Epidemic, Lipid profile, Coronary heart disease

## Abstract

**Background:**

Obesity has become global epidemic in the last three decades, whereas Coronary Heart Disease (CHD) still remains the most important cause of mortality in the world. The study was aimed at determining the pattern of lipid profile for the obese and CHD population in Pakistan. As obesity is a strong predisposing risk factor for CHD, we aimed to analyze the lipid parameters in both conditions and compare them with the healthy controls of the same ethnicity.

**Methods:**

Blood samples were collected from one thousand individuals (500 with CHD, 250 with obesity, 250 healthy controls). The lipid profile (total Cholesterol, triglycerides, HDL-C, LDL-C and VLDL) was measured using commercially available kits. The pattern of dyslipidemia was then studied by comparing the results in both groups with controls as well as population cutoffs. The quantitative variables were checked for normality and log transformation was done for variables where appropriate. Analysis of variance and logistic regression were done to check the association of lipid parameters with obesity and CHD.

**Results:**

The obese and CHD groups showed a dyslipidemic profile than the healthy controls. CHD group had a higher proportion of CHD in any of the first degree blood relatives (36.0% vs. 1.8%), a similar trend was observed in the obese group, where 63.9% cases had positive family history. Among cases, 50.7% had combined lipid abnormalities, i.e., the values of TC, LDL-C, TG and HDL-C, all were deranged. Whereas 49.52% had TC more than normal cut off (> 200 mg/dl), 51.6% had LDL-C > 100 mg/dl. Similarly, 80.4% of patients had TG levels more than upper normal range (> 150 mg/dl) and 64% had HDL values in moderate CHD risk group (< 50 mg/dl). The results show that Pakistani cases are hyperlipidemic for lipid traits except for HDL which is lowered. Patients with comorbidities also had lipid profiles deviated from the normal range.

**Conclusion:**

The study provides information regarding the aberration of lipid profile in the metabolic disorders that can increase the predisposition to complications.

## Introduction

Obesity is defined as having an excess of body weight. A World Health Organization (WHO) release defined obesity as a chronic disease increasing globally replacing traditional health concerns [1]. It is directly related to cardiovascular problems and children whose parents are cardiovascular patients tend to have higher weight in the childhood and develop obesity as adults [2]. Coronary heart disease (CHD) has been estimated to become the leading cause of death in developing countries by 2020 [3]. More than 80% of global CHD burden occurs in low income countries but the knowledge regarding important risk factors is derived largely from developed countries. There is geographic variability in the prevalence of CHD risk factors [4] and it is important to use the local data when discussing the relation between a disease and its risk factors,. It is still unclear to what extent the CHD risk factors known in the western civilizations are applicable in Pakistan. One possible reason is the high prevalence of CHD risk factors like hypertension, obesity and dyslipidemia in Pakistan [5]. Considering obesity as CHD risk factor, it has been shown that increased abdominal obesity, insulin resistance and type 2 diabetes (T2DM) are more prevalent in South Asians and are important CHD risk factors [6]. As CHD can be a sequel of obesity, the biochemical pathways involved in the development of obesity have also been investigated to play a role in the development of CHD [7].

Blood lipid levels are modifiable risk factors for atherosclerosis and CHD. Being hydrophobic in nature, cholesterol, cholesterol esters, triglycerides and phospholipids are transported to the other tissues in the form of lipoproteins. Major classes of lipoproteins are chylomicrons (CM), low density lipoproteins (LDL) and high density lipoproteins (HDL), named by the site of their assembly and type of lipid and apo protein they have [8]. Excess fatty acids (FA) in the liver are converted into triacyglycerols which along with phospholipids, free and esterified cholesterol are packaged into very low density lipoprotein (VLDL) along with a variety of apo proteins. While travelling through the peripheral tissues, triacyglycerol content is hydrolysed with the help of lipoprotein lipase (LPL) into FA and VLDL remnants [9]. VLDL remnants through further hydrolysis of triglyceride contents give rise to intermediate density lipoproteins (IDL) and LDL. LDL having apoB100 apo protein component is the major cholesterol carrier in peripheral circulation [10]. Elevated plasma levels of these non HDL lipoproteins are major CHD risk factors [11].

It has been observed that many lipid/lipoprotein abnormalities are prevalent in obesity and heart problems, collectively termed as dyslipidemia, however, these dyslipidemias are often hyperlipidemia wherein majority of lipids are shifted towards the upper limits of range or higher than the range. Owing to the recent modernization of the lifestyle and availability of transportation means combined with a unique ethnicity have resulted in the high prevalence of metabolic disorders in Pakistan like the rest of the world. This not only affects the daily activities, work performance and social interactions but also poses a huge burden on healthcare. Keeping in view the importance of lipid traits in the development of nutritional disorders, the current study was conducted to investigate the lipid profile patterns in the obese and CHD patients in Pakistani subjects.

## Materials and methods

### Study design, subjects selection and evaluation criteria

The study followed a case-control study design and was performed at the Department of Microbiology and Molecular Genetics, University of the Punjab, Lahore. It included 500 CHD patients, 250 obese and 250 age and sex matched controls. The patients were recruited from various hospitals of Punjab. The patients come from not only various cities and villages of Punjab but also from all over Pakistan to these government hospitals, therefore the samples were representative of the majority of the population. The selected CHD subjects had suffered from a non-fatal myocardial infarction with diagnosis made by the consultant cardiologist based on the reports of ECG, cardiac echo, angiography, troponine T/I and clinical history. Only those CAD cases were selected which were recently diagnosed and had not started lipid lowering or antihypertensive drugs therapy.. The inclusion criteria for obese subjects was based on BMI cut offs defined for Asian populations previously (BMI > 23Kg/m^2^ as overweight and > 26Kg/m^2^ as obese) [12, 13]. Waist to hip ratio was used as an indirect indication of upper and lower body fat distribution [14].. The controls were apparently healthy subjects having BMI 18 and 23 and not having any CHD history. No underweight subject, pregnant women, persons with malignancies/infections were included in the study, also CHD subjects with obesity were excluded. The presence of comorbidities (diabetes, hypertension) was identified. Diabetes was indicated by fasting blood glucose (FBG) of 6.7 mmol/L or above or a 2 h postprandial blood glucose of 11.1 mmol/L or above. The criteria for classification of hypertensive and normotensive individuals was based on report from seventh Joint National Committee for Hypertension (JNC-V) [15]. Blood pressure measurements were taken by a trained health professional after the subject had a 5 min sitting. The study was approved by the institutional ethical committee (Ethical Committee, School of Biological Sciences, University of the Punjab).

### Sample collection

5 ml overnight fasting blood were collected in a vacutainer contining gel clot activator from the median cubital vein with tourniquet tied to the limb and fingers squeezed. Blood was centrifuged for 10 min at 10,000 rpm to settle all the formed elements and separate serum. Serum was transferred to an autoclaved eppendorf and were immediately frozen until further analyses.

### Lipid profile estimation

Plasma lipids and lipoprotein variables, namely total cholesterol (TC), and triglycerides (TG), were determined using commercially available kits (Spectrum Diagnostics). All optical density measurements were made using Epoch Biotek (SN 257866, USA) at 546 nm for TC and TG. High density lipoprotein cholesterol (HDL-C), and low density lipoprotein cholesterol (LDL-C) were measured according to Mohsen Ibrahim et al [16]. HDL determination required precipitation of very low density lipoprotein (VLDL) and low density lipoprotein (LDL) particles first from serum, while what is left in serum is HDL-C which is then detected on the same principle as total cholesterol. LDL-C determination makes use of a modified polyvinyl sulfonic acid (PVS) and polyethylene-glycol methyl ether (PEGME) coupled classic precipitation method. PVS and PEGME react with LDL, VLDL, and chylomicron (CM) resulting in inaccessibility of these particles by cholesterol oxidase (CHOD) and cholesterol esterase (CHER), whereas HDL reacts with the enzymes. Addition of a second detergent containing reagent releases LDL from the PVS/PEGME complex. The released LDL then reacts with the enzymes to produce H_2_O_2_ which is quantified by the Tinder reaction.

### Statistical analysis

Data was analyzed for means and standard deviation for all parameters. Data analysis was done by Microsoft excel and statistical package for social sciences (SPSS version 22). The lipid profile parameters were checked for normality. The association of lipid profile parameters with the CHD/Obese status was checked using binary logistic regression analysis using CHD/obese status as dependent and lipid traits as independent variables. The one-way analysis of variance (ANOVA) was done to compare the means of different groups. The analyses were adjusted for confounders including age, gender, socioeconomic status (SES), the hypertensive and diabetic status while analyzing the association with CHD and obese status. A *p*-value < 0.05 was considered statistically significant for all analyses.

## Results

In total, one thousand subjects were included in the study. The mean age of CHD group was 59.1 ± 12.7, for obese group 39.63 ± 15.19 and for the controls 55.8 ± 10.3. The demographic characteristics of controls, and obese and CHD cases are summarized in Table 1.

**Table 1:** Baseline characteristics of the population under study. Categorical variables are expressed in number in percentage; *p* is the statistically significant value

Variables	Cases		Controls	***p***-value CHD vs Controls	***p***-value Obese vs Controls
	CHD	Obese			
**Number**	500	250	250	–	–
**Age**	59 ± 12.7	39.6 ± 15.19	55.87 ± 10.37	0.002	3.2 × 10^−57^
**Gender**					
**Males (%)**	58.2	54	55.4	0.49	0.131
**Females (%)**	41.8	46	44.6		
**Diabetic (%)**	64.3	32	13.4	< 0.0001	< 0.0001
**Hypertension (%)**	59.9	25.6	16.2	< 0.0001	< 0.0001
**Smoking (%)**	29.4	11.9	10.3	< 0.0001	< 0.0001
**Family history (%)**	36.0 (FH of CHD)	63.9 (FH of Obesity)	1.8 (CHD) 8.4 (Obesity)	< 0.0001	< 0.0001
**BMI (Kg/m**^**2**^**)**	22.4 ± 6.7	36.9 ± 6.4	21.4 ± 9.1	< 0.0001	< 0.0001
**WHR**	0.85 ± 0.06	1.00 ± 0.09	0.85 ± 0.10	1	< 0.0001

As far as the blood lipid levels are concerned, patients were more dyslipidemic than the healthy controls. Mean TC, TG, high density lipoprotein cholesterol (HDL-C), low density lipoprotein cholesterol (LDL-C), and TC/HDL ratios are shown in the Table 2. The mean TC, TG and LDL-C were significantly higher and HDL-C was significantly lower in the cases as compared to the controls. The TC/HDL-C ratio, which is considered to be a sensitive indicator of cardiovascular disease risk, was also higher in the cases, obese as well as CHD, than the controls, and the difference was statistically significant. CHD had also association with a history of CHD in any of the first degree blood relatives in the family (36.0% vs. 1.8%), a similar trend was observed in the obese group where 63.9% cases had a family history of obesity.

**Table 2:** Blood lipid levels of the patients and controls under study

Variable	CHD	Obese	Controls	***p***-value CHD vs Controls^a^	***p***-value Obese vs Controls^b^	***p***-value ANOVA^c^
**TC (mg/dl)**	208.2 ± 54.11	214.4 ± 35.02	158.6 ± 33.14	2.9 × 10^−23^	2.4 × 10^−36^	< 0.001
**TG (mg/dl)**	214.5 ± 74.60	265.1 ± 50.38	187.7 ± 41.75	2.09 × 10^− 34^	1.8 × 10^−7^	< 0.001
**HDL-C (mg/dl)**	45.1 ± 11.63	43.1 ± 4.23	80.6 ± 12.71	1.06 × 10^−71^	1.6 × 10^− 179^	< 0.001
**LDL-C (mg/dl)**	104.6 ± 37.94	115.7 ± 21.99	78.1 ± 15.42	1.6 × 10^−34^	1.3 × 10^− 24^	< 0.001
**TC/HDL-C**	4.6 ± 0.87	5.0 ± 0.97	2.7 ± 1.11	1.6 × 10^−34^	3.8 × 10^−98^	< 0.001

Continuous variables are expressed in mean ± SD

*TC* total cholesterol level, *VLDL* very low density lipoprotein concentration, *LDL-C* low density lipoprotein cholesterol level, *HDL-C* high density lipoprotein level and TG: triglycerides level

^a^indicates the significance of difference between the means of CHD vs controls

^b^indicates the significance of difference between the means of Obeses vs controls

^c^indicates the significance of difference between means of all groups as tested by ANOVA

In the cases, the proportion of males suffering from the disease was higher than females (57% vs 42.8%). The mean age was slightly higher in female patients than male patients (59.5% vs 58.5%). The percentage of diabetes was also higher in males than females. However, among CHD females, the ratio of hypertension was more than males and family history of having CHD was also higher in females than male patients. However, these differences were not statistically significant; the only significant difference between male and female patients was their smoking status, which was present almost exclusively in males (50%) and only 0.6% females were smokers ang CHD whereas in the obese group, the proportion of smokers was low compared to the CHD group as well as smoking did not appear to be associated with obesity.

Lipid abnormalities were present individually as well as in combination. Among the cases (CHD and obese combined), 50.7% had combined lipid abnormalities, i.e., the values of TC, LDL-C, TG and HDL-C were all deranged. Whereas 49.5% had TC more than normal cut off (> 200 mg/dl), 51.6% had LDL-C > 100 mg/dl. Similarly, 80.4% of patients had TG levels more than upper normal range (> 150 mg/dl) and 64% had HDL values in moderate CHD risk group (< 50 mg/dl) (Table 3).

**Table 3:** Genderwise presentation of subjects in the CHD and obese group showing deranged Lipid Profile

Variables (mg/dl)	CHD		Obese	
	Males	Females	Males	Females
**TC > 199**	51.5% (124)	50.3% (87)	44.7% (114)	45% (99)
**TG > 149**	78.8% (190)	82.1% (142)	86.6% (221)	85% (187)
**HDL-C < 40**	45.5% (110)	53.8% (93)	48.2% (123)	53.6% (111)
**< 49**	65.6% (158)	55.1 (96)	50.9% (130)	54.9 (114)
**LDL-C > 99**	50.6% (122)	56.1% (97)	54.1% (138)	50.4% (118)

The comparison of the means of the lipid profile parameters between males and females turned out that among the CHD males/females as well as obese males/females, no significant difference was observed (Table 4). However, the logistic regression analysis of all the parameters with CHD/obese status turned out to be highly significant (Table 5).

**Table 4:** Lipid profile values of the Cases (CHD & obese) separated by their gender status

Variable	CHD		***P*** value	Obese		***P*** value
	Males	Females		Males	Females	
**TC (mg/dl)**	208.5 ± 54.6	207.7 ± 53.5	0.88	199.5 ± 45.1	192.2 ± 43.4	0.074
**TG (mg/dl)**	213.2 ± 75.6	216.2 ± 73.2	0.69	237.6 ± 67.1	226.2 ± 62.9	0.056
**HDL-C (mg/dl)**	44.2 ± 11.2	46.3 ± 12.1	0.07	55.1 ± 17.3	54.7 ± 18.0	0.839
**LDL-C (mg/dl)**	104.8 ± 39.9	104.2 ± 35.1	0.87	103.7 ± 29.2	98.7 ± 27.4	0.056

**Table 5:** Logistic regression model for CHD/Obese status

Variable	CHD		Obese	
	OR (95% CI)	***p-***value	OR (95% CI)	***p***-value
**TC (mg/dl)**	2.18 (1.76–2.71)	< 0.0001	1.02 (1.01–1.03)	< 0.0001
**TG (mg/dl)**	1.42 (1.19–1.69)	< 0.0001	1.113 (1.09–1.136)	< 0.0001
**HDL-C (mg/dl)**	7.07 (5.14–9.71)	< 0.0001	0.814 (0.778–0.853)	< 0.0001
**LDL-C (mg/dl)**	2.79 (2.22–3.50)	< 0.0001	1.037 (1.02–1.05)	< 0.0001

## Discussion

Abnormalities in the lipid profile, specifically hypertriglyceridemia and low levels of HDL-C have been shown to be a strong predisposing issue to many diseases including obesity, diabetes and cardiovascular diseases. It has been estimated that the risk of CVD decreases by 2 to 3% for every 1 mg/dL increase in HDL-C [17]. Despite some controversy, elevated levels of triglycerides, fasting as well as nonfasting, also appear to be an independent risk factor for CHD [18, 19]. Evidence from epidemiologic studies suggests that the co-occurrence of low HDL-C and elevated triglyceride levels is a strong risk factor for CHD [20, 21] while posthoc analyses of several studies have shown that patients with low HDL-C and elevated triglycerides have the highest rate of major coronary events [22]. Whether an increased level of small dense LDL represents an independent risk factor remains somewhat controversial, but it is clearly associated with an increase in CHD risk [23]. Serum cholesterol has been shown to be an established CHD risk factor in European people [24] as well as Asians [25] Elevated levels of plasma LDL-C are major CHD risk factors as therapy with LDL-C drugs have reduced CHD risk and the reduction is proportion to decrease in LDL-C levels [26, 27].

As expected, already established obesity and CHD risk factors had a high prevalence in the patients in the current study as well. It was clear that lipid profile of patients was more atherogenic and patients exhibited significantly higher prevalence of other CHD risk factors like hypertension, DM and smoking compared to age and sex matched controls. The findings are in accordance with another Pakistani study and the studies carried out in other developed countries [28–30].

High percentage of males was found to be suffering from the disease than females. This does not seem to be by chance, rather shows increased prevalence of the disease among males in the selected study group as well as the general population. Similar results were seen in another study conducted in Pakistan [31]; however the reports of some other researchers [5, 32] were contradictory. Secondary causes, such as high values for mean TC, LDL-C, smoking rate, DM and low values for HDL-C in males than females may be due to higher prevalence in males than females. This may also lead to altered lipid profile between females and males.

Hypercholesterolemia, either genetic or acquired, is an independent CHD risk factor. It is estimated that 56% of the total heart diseases may be due to hypercholesterolemia (> 200 mg/dl) alone [31]. It was observed that patients had significantly higher TC levels, also hypercholesterolemia was heterogeneous within patients as hypertensive males were having high cholesterol levels than normotensive (*p* = 0.006) and diabetic females were having high cholesterol than non diabetic (*p* = 0.00004). Non diabetic females were having the lowest cholesterol levels among cases. This indicates that cholesterol levels are also being affected by diabetes and the lipid profile is more disturbed in subjects who have other metabolic conditions. Many authors have reported the lipid levels to be similar among Asian and western countries and attributed the increased risk of CHD to be due to insulin resistance and central obesity [4, 33, 34].

TC/HDL ratio is considered to be a sensitive predictor of cardiovascular disease risk, especially if the values are ≥6 [35–37]. Although in our study, the TC/HDL ratio is significantly higher than controls, the values are below this cutoff. In the USA, however, the TC / HDL ratio above 5 is considered to be atherogenic. Interestingly, using this cutoff, we noticed a gender difference in the patterns of dyslipidemia among diabetic and non diabetic groups. Among diabetics, the mean ratio for males, 5.22 is higher than the USA cut off while females had a ratio, 4.88 below the cut-off. On the contrary, in the non-diabetic group, females had a ratio, 5.08, higher than the cutoff while males had a mean ratio, 4.89, lower than the cutoff.

In the current study, increased TG concentrations are consistently accompanied by low HDL concentrations that often coexists with the elevated plasma glucose levels because high amount of sugar in plasma (hyperglycemia) results in the transfer of cholesterol esters from HDL-C to VLDL particles [38]. Further lowering in HDL concentration results from its conversion by hepatic lipase into smaller particles which are rapidly cleared from plasma [39]. Resulting VLDL particles form cholesteryl ester depleted small dense LDL-C particles that are taken up by arterial wall macrophages causing atherogenesis [40].

The study had limitation of small sample size. The sample size of one thousand is not sufficient to rule out any minor gender related differences. The coexistence of several conditions (obesity, diabetes, hypertension, cardiovascular diseases) further complicates the analyses. Despite these limitations, the study gives basic information about dyslipidemia in obese and CHD population in Pakistan and can be helpful in advanced research about lipid profile and establishing a correlation between lipid profile parameters and dyslipidemia associated abnormalities in Pakistani population.

## Conclusion

The study showed that dyslipidemia is an important factor contributing to the development of obesity and CHD in Pakistan. The study provides information regarding the importance of considering the lipid profiling in the treatment of these metabolic disorders. Apparently healthy individuals should get their lipid profiling done once or twice a year and go for appropriate lifestyle changes so as to prevent the onset of metabolic aberrations. The recent westernized lifestyle in the Pakistani youth combined with the inherent tendency of fat deposition in the abdominal area has increased the risk of obesity and its sequelae. In these situations, a simple lipid profile test can help manage the dyslipidemia before the onset of disease.

## Data Availability

All the necessary information has been provided along with the manuscript, however, the corresponding author can be contacted for any information related to this paper.

## Abbreviations

CHD: Coronary heart disease
HDLC: high-density lipoprotein cholesterol
LDL-C: Low-density lipoprotein cholesterol
TC: Total cholesterol; TG: Triglycerides
WHO: World Health Organization
LPL: lipoprotein lipase
JNC-V: Joint National Committee for Hypertension
